# Association between urinary biomarkers of polycyclic aromatic hydrocarbons and severe abdominal aortic calcification in adults: data from the National Health and Examination Nutrition Survey

**DOI:** 10.1186/s12872-023-03122-0

**Published:** 2023-02-23

**Authors:** Xi Yang, Haobin Zhou, Hao Zhang, Peijian Zhang, Zhikang Zheng, Dingli Xu, Qingchun Zeng

**Affiliations:** 1 State Key Laboratory of Organ Failure Research, Department of Cardiology, Nanfang Hospital, Southern Medical University, 1838 Northern Guangzhou Ave, Guangzhou, 510515 China; 2 Department of Cardiology, The First Affiliated Hospital of Guangzhou Medical University, Guangzhou, 510120 Guangdong China; 3 Cardiovascular Medicine Department, Sun Yat-Sen Memorial Hospital, Sun Yat-Sen University, Guangzhou, 510120 China

**Keywords:** Abdominal aortic calcification, PAHs exposure, NHANES, Logistic regression

## Abstract

**Objective:**

Recent studies have found that polycyclic aromatic hydrocarbons (PAHs) exposure may increase the risk of cardiovascular disease. The present study aimed to explore the association between PAHs exposure and severe abdominal aortic calcification (AAC) in adults.

**Methods:**

Data were collected from the 2013–2014 National Health and Nutrition Examination Survey. PAHs exposure was analyzed from urinary mono hydroxylated metabolites of PAHs. Logistic regression models and subgroup analysis were performed to explore the association of PAHs exposure with severe AAC prevalence.

**Results:**

A total of 1,005 eligible individuals were recruited into the study. After adjusting for confounding factors, those with the highest quartiles of 1-hydroxynaphthalene (1-NAP: OR 2.19, 95% CI 1.03–4.68, *P*_for trend_ < 0.001), 2-hydroxynaphthalene (2-NAP: OR 2.22, 95% CI 1.04–4.64, *P*_for trend_ < 0.001) and 1-hydroxypyrene (1-PYR: OR 2.15, 95% CI 1.06–4.33, *P*_for trend_ < 0.001) were associated with an increased prevalence of severe AAC in the adults compared to those who in the lowest quartile.

**Conclusion:**

This study found that urinary 1-NAP, 2-NAP and 1-PYR were positively associated with severe AAC prevalence in adults.

## Introduction

Abdominal aortic calcification (AAC) is an early manifestation of atherosclerosis in abdominal aortic wall [1]. AAC has been proved to be a predictor of cardiovascular mortality, poor prognosis, and all-cause mortality [2–4]. Aortic calcification was once considered as a pathological disease with passive deposition of calcium and phosphate in the vascular wall, which is related to degeneration [5]. In recent years, increasing studies have shown that aortic calcification is an active and highly adjustable tissue mineralization process involving cadmium exposure [6], metabolic syndrome [7], inflammatory infiltration [8] and lead burden [9]. However, severe AAC still plagues the aging population. Therefore, identifying factors that can be improved may be of great significance for early intervention to delay or block the progress of AAC.

Polycyclic aromatic hydrocarbons (PAHs) are a group of existing emerging contaminants produced by incomplete combustion of organic materials [10]. PAHs released into the environment from coal and oil combustion [11], road transportation [12], waste incineration [13] and food cooking [14], and got into the body through inhalation, ingestion or dermal exposure. PAHs are metabolized in the liver through cytochrome P450 (CYP) enzymes and readily eliminated in urine after entering the body [15]. Naphthalene, fluorene, phenanthrene and pyrene in urine are PAH metabolites and considered as biomarkers to evaluate the recent exposure of PAHs [16, 17]. Substantial observational studies and randomized trials revealed the potential role of PAHs in increasing ischemic heart disease [18], lung cancer [19], neurological disorders [20], attention deficit hyperactivity disorder [21], anxious/depressed and attention problems [22].

PAHs exposure have been demonstrated to increase the risk of hypertension, peripheral arterial disease (PAD), coronary heart disease (CAD), myocardial infarction (MI) and stroke. Abdominal aortic calcification (AAC), an early manifestation of abdominal aortic wall atherosclerosis driven by inflammation. An open randomized crossover trial found that reducing personal exposure to air pollution potentially reduce symptoms and improve a range of cardiovascular health measures in patients with coronary heart disease.

However, the relationship between PAHs exposure and severe AAC risk is currently lacking. Evaluating the relationship between PAHs exposure and severe AAC can improve our understanding of the impact of PAHs exposure on vascular calcification from a chemical contaminant’s perspective, and provide insights into the prevention and management of vascular calcification progression.

## Methods

### Study population

The National Health and Nutrition Examination Survey (NHANES) is a cross-sectional survey based on population to collect information of the health and nutritional status in American children and adults. This survey includes health interviews and health examinations, the former was conducted in participants’ homes and the latter was executed in a mobile examination center (MEC). This database can be found in online repositories with data released every 2 years. Urinary mono hydroxylated metabolites of PAHs (OH-PAHs) were measured in a one third subsample of persons 6 years and older. The method details of sampling and data collection have been presented in previous study [23].

Because only 2013–2014 NHANES database contained both data of PAHs and abdominal aortic calcification score (AACs), we extracted data from this survey cycle to carry out this research. Participants aged < 40 years old were excluded for dual-energy X-ray absorptiometry (DXA) scan so that lack of the data of AACs in NHANES database. We extracted the demographic information, clinical information, laboratory and physical exam results of 10,175 participants from 2013–2014 NHANES database. Participants aged < 40 years old (n = 6,360), missing the data of PAHs (n = 2,652), or missing the AAC data (n = 158) were excluded and 1,005 participants were recruited into final analysis. The NHANES protocol were approved by the NCHS Ethics Review Board, and all enrolled participants provided written informed consent.

### Quality assurance and quality control

The NHANES quality assurance and quality control (QA/QC) protocols meet the 1988 Clinical Laboratory Improvement Act mandates. Detailed QA/QC instructions are discussed in the NHANES Laboratory/Medical Technologists Procedures Manual (LPM). A detailed description of the quality assurance and quality control procedures can be found on the NHANES web site.

### Measurement of urinary OH-PAHs

PAHs were available in the “Laboratory data- Polycyclic Aromatic Hydrocarbons in Urine dataset”. Based on the method of derivatization and gas chromatography-tandem mass spectrometry [24], the samples of each subject were analyzed for OH-PAHs. In the present study, data on seven OH-PAHs were available from the 2013–2014 NHANES survey cycles for analysis, including 1-hydroxynaphthalene (1-NAP), 2-hydroxynaphthalene (2-NAP), 2-hydroxyfluorene (2-FLU), 3-hydroxyfluorene (3-FLU), 1-hydroxyphenanthrene (1-PHE), 2-hydroxyphenanthrene & 3-hydroxyphenanthrene (2&3-PHE), and 1-hydroxypyrene (1-PYR). Urine samples were measured OH-PAHs by using enzymatic deconjugation, followed by on-line solid phase extraction, and separation and quantified by isotope dilution high performance liquid chromatography-tandem mass spectrometry. To control the differences from urine dilution, all samples were measured the levels of specific gravity by using a handheld refractometer (Urine-Specific-Gravity-Refractometer-PAL-10-S-P14643C0; TAGO USA, Inc. Bellevue, WA 98005 USA). The analytes of OH-PAHs (ng/L) measured in urine, divided by the urine creatinine level (mg/dL) multiplied by 0.01 and expressed as nanograms per gram of creatinine, were corrected for creatinine concentration to reduce the variability induced by urine volume differences.

### AAC evaluation

According to the methods presented in Kauppila score system, AAC score was quantified by lateral lumbar spine images obtained from dual-energy X-ray absorptiometry (DXA, Densitometer Discovery A, Hologic, Marlborough, MA, USA) and was used for AAC evaluation. DXA scans exclude participants who were weighed over 450 pounds, aged < 40 years, pregnant or used barium in the past 7 days. Evaluating abdominal aortic calcification was assessed at the anterior and the posterior walls of the abdominal aorta adjacent to vertebrae L1–L4. Kauppila scores are ranged from 0 to 6 for each segment and 0 to 24 for the total AACs [25]. Higher AACs reflects a more serious abdominal aortic calcification. Previous research has demonstrated that severe AAC was identified as a total score > 6 which represented significant aortic calcification [26, 27].

### Covariates

Sociodemographic factors of age, gender, race, education level, alcohol use and smoking status were collected with standardized questionnaires from household interviews. Body weight, height, and blood pressure of participants were measured in physical examinations at MEC. Plasma glucose, cotinine, total cholesterol (TC) and triglycerides were processed and measured according to laboratory methods [28]. Race was classified into Mexican American, other Hispanic, non-Hispanic White, non-Hispanic Black, and other races. Family income was categorized as < $20,000, $20,000–$55,000, $55,000–$75,000, and ≥ $75,000. Education level was classified as less than high school, high school or equivalent, or college and above. Smoking status was grouped into never smokers, current smokers and former smokers. Patients who reported never smoked or less than a hundred cigarettes in their lifetime were defined as never smokers. Patients who reported smoking more than a hundred cigarettes in their lifetime and still smoking currently were defined as current smokers. Patients who reported had smoked more than a hundred cigarettes in their lifetime but not smoking currently were defined as former smokers. Body mass index (BMI) is a person’s weight in kilograms (kg) divided by height in meters squared (m^2^). The diagnosis of diabetes is based on HbA1c ≥ 6.5%, a fasting blood glucose level of ≥ 7.0 mmol/l, using current antidiabetic therapy, or self-reported diabetes diagnosis. Hypertension was defined as systolic blood pressure ≥ 130 mmHg, diastolic blood pressure ≥ 80 mmHg, or currently taking hypertension drugs, or self-reported hypertension diagnosis.

### Statistical analysis

Continuous variables were presented as mean and standard deviation (SD). Numbers and percentages were employed to express categorical data. Differences between groups were tested by the χ^2^ test for categorical data, and student’s t-test for continuous data. Logistic regression models were used to assess the association between PAHs and severe AAC prevalence. Model 1 was unadjusted. Model 2 was adjusted for gender, age, and race. Model 3 included the variables in model 2 and BMI, education, smoking category, alcohol use, family income, hypertension, diabetes, triglycerides, TC and cotinine levels. PAHs was categorized by quartile, with the lowest quartile used as the reference. The ORs and 95% CIs were calculated. Subgroup analyses were performed for PAHs and severe AAC prevalence by the following variables: gender, age (< 60 years and ≧ 60 years), BMI (< 25, 25–30 and > 30), race, diabetes and hypertension. The probabilities here were all two-sided with significance at *P* < 0.05. All analyses were conducted using R version 3.3.3.

## Results

### Baseline characteristics of participants

**Fig. 1:**
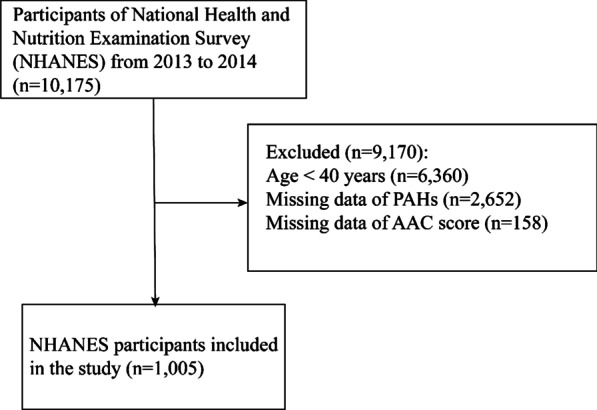
Flow chart of the screening process for the selection of eligible NHANES participants

A detailed flow chart (Fig. 1) depicted participant selected from the NHANES 2013–2014 database. We extracted data of 10,175 participants from 2013–2014 NHANES database and analyzed 1,005 participants with complete AAC and PAHs data in this study, of whom with severe AAC were non-Hispanic white, more likely to be highly educated, a current smoker, to earn a medium income, to have a higher prevalence of diabetes (Table 1).

**Table 1:** Characteristics of participants by different AACs levels, NHANES 2013–2014^a^

Characteristics	AACs categories		
	AACs ≤ 6	AACs > 6	*P* value
Age, years	57.88 (10.84)	69.33 (9.31)	< 0.001
Race, No. (%)			0.033
Mexican American	127 (13.89)	11 (12.09)	
Other Hispanic	88 (9.63)	3 (3.30)	
Non-Hispanic White	394 (43.11)	50 (54.95)	
Non-Hispanic Black	185 (20.24)	11 (12.09)	
Other race	120 (13.13)	16 (17.58)	
Gender, No. (%)			0.413
Male	441 (48.25)	48 (52.75)	
Female	473 (51.75)	43 (47.25)	
Educational levels, No. (%)			0.439
Less than high school	100 (10.95)	9 (9.89)	
High school or equivalent	310 (33.95)	37 (40.66)	
College or above	503 (55.09)	45 (49.45)	
Smoking status, No. (%)			< 0.001
Current	334 (36.54)	59 (64.84)	
Former	260 (28.45)	17 (18.68)	
Never	320 (35.01)	15 (16.48)	
Income, No. (%)			0.005
< 20, 000	163 (18.84)	20 (22.73)	
20, 000–55, 000	262 (30.29)	40 (45.45)	
55, 000–75, 000	165 (19.08)	11 (12.50)	
> 75, 000	275 (31.79)	17 (19.32)	
Hypertension, No. (%)	479 (52.41)	45 (49.45)	0.590
Diabetes, No. (%)	437 (47.81)	74 (81.32)	< 0.001
Body mass index, kg/m^2^	28.77 (5.66)	27.09 (4.28)	0.006
Total cholesterol, mg/dL	197.56 (44.93)	186.42 (39.26)	0.028
Triglycerides, mg/dL	125.74 (88.89)	115.44 (54.03)	0.466
Cotinine, ng/mL	60.10 (143.62)	89.21 (240.93)	0.276
Alcohol, drinks/day	1.40 (1.83)	1.44 (1.62)	0.829
1-NAP, ng/g	4.48 (4.54)	23.97 (22.67)	< 0.001
2-NAP, ng/g	1.21 (1.04)	3.77 (3.12)	< 0.001
3-FLU, ng/g	0.31 (0.82)	0.59 (1.18)	0.034
2-FLU, ng/g	0.32 (0.43)	0.54 (0.97)	0.038
1-PHE, ng/g	0.31 (0.77)	0.56 (0.78)	0.004
1-PYR, ng/g	0.34 (0.65)	1.63 (1.89)	< 0.001
2 & 3- PHE, ng/g	0.30 (0.51)	0.48 (0.69)	0.018

Categorical was expressed as numbers and percentages

AACs, abdominal aortic calcification score; BMI, body mass index; 1-NAP, 1-hydroxynaphthalene; 2-NAP, 2-hydroxynaphthalene; 3-FLU, 3-hydroxyfluorene; 2-FLU, 2-hydroxyfluorene; 1-PHE, 1-hydroxyphenanthrene; 1-PYR, 1-hydroxypyrene; 2 & 3-PHE, 2 & 3-hydroxyphenanthrene

^a^Data are presented as mean (SD) unless otherwise indicated

### High urinary OH-PAHs are associated with increased risk of severe AAC

The associations between the urinary OH-PAHs and severe AAC are displayed in Table 2. In univariate logistic regression, participants in the highest quartile of 1-NAP, 2-NAP and 1-PYR exposure showed a higher risk of severe AAC compared to those who in the lowest quartile. These associations remained significant after adjustment for age, gender and race (model 2). In fully adjusted models (model 3), compared to the lowest quartile, those with the highest quartiles of 1-NAP (OR 2.19, 95% CI 1.03–4.68, *P*_for trend_ < 0.001), 2-NAP (OR 2.22, 95% CI 1.04–4.64, *P*_for trend_ < 0.001) and 1-PYR (OR 2.15, 95% CI 1.06–4.33, *P*_for trend_ < 0.001) showed a higher risk of severe AAC.

**Table 2:** ORs and 95% CIs for sever AAC according to urinary OH-PAHs, NHANES 2013–2014

Urinary OH-PAHs	Odds Ratio (95% Confidence Interval)			
		Model 1	Model 2	Model 3
1-NAP	Continuous	1.07 (1.05, 1.09)	1.07 (1.05, 1.09)	1.07 (1.04, 1.10)
	Quartile 1	1.00 (reference)	1.00 (reference)	1.00 (reference)
	Quartile 2	1.05 (0.61, 1.79)	1.02 (0.60, 1.74)	1.01 (0.45, 2.30)
	Quartile 3	1.74 (1.07, 2.85)	1.65 (1.01, 2.70)	1.36 (0.63, 2.89)
	Quartile 4	2.35 (1.45, 3.80)	2.23 (1.37, 3.60)	2.19 (1.03, 4.68)
	*P* for trend	< 0.001	< 0.001	< 0.001
2-NAP	Continuous	1.42 (1.29, 1.57)	1.40 (1.27, 1.55)	1.35 (1.17, 1.57)
	Quartile 1	1.00 (reference)	1.00 (reference)	1.00 (reference)
	Quartile 2	1.27 (0.76, 2.13)	1.26 (0.75, 2.11)	1.13 (0.52, 2.50)
	Quartile 3	1.38 (0.83, 2.29)	1.44 (0.87, 2.41)	1.26 (0.58, 2.81)
	Quartile 4	2.44 (1.52, 3.93)	2.48 (1.54, 4.00)	2.22 (1.04, 4.64)
	*P* for trend	< 0.001	< 0.001	< 0.001
3-FLU	Continuous	1.13 (0.96, 1.32)	1.14 (0.97, 1.34)	1.11 (0.90, 1.37)
	Quartile 1	1.00 (reference)	1.00 (reference)	1.00 (reference)
	Quartile 2	0.91 (0.55, 1.49)	0.90 (0.55, 1.48)	0.89 (0.41, 1.93)
	Quartile 3	1.10 (0.68, 1.78)	1.07 (0.66, 1.74)	1.01 (0.47, 2.18)
	Quartile 4	1.35 (0.85, 2.15)	1.38 (0.87, 2.20)	1.46 (0.66, 3.25)
	*P* for trend	0.136	0.115	0.346
2-FLU	Continuous	1.32 (0.99, 1.75)	1.30 (0.98, 1.72)	1.13 (0.76, 1.69)
	Quartile 1	1.00 (reference)	1.00 (reference)	1.00 (reference)
	Quartile 2	0.79 (0.49, 1.27)	0.93 (0.59, 1.47)	0.76 (0.37, 1.51)
	Quartile 3	0.83 (0.51, 1.33)	0.70 (0.44, 1.35)	0.65 (0.30, 1.39)
	Quartile 4	1.10 (0.70, 1.73)	1.08 (0.68, 1.13)	0.78 (0.38, 1.59)
	*P* for trend	0.051	0.071	0.562
1-PHE	Continuous	1.15 (0.97, 1.37)	1.16 (0.97, 1.39)	1.11 (0.81, 1.49)
	Quartile 1	1.00 (reference)	1.00 (reference)	1.00 (reference)
	Quartile 2	0.86 (0.52, 1.41)	0.87 (0.53, 1.44)	0.87 (0.40, 1.88)
	Quartile 3	1.07 (0.66, 1.72)	1.07 (0.66, 1.73)	0.99 (0.46, 2.19)
	Quartile 4	1.50 (0.95, 2.37)	1.42 (0.90, 2.25)	1.38 (0.67, 2.87)
	*P* for trend	0.115	0.104	0.512
1-PYR	Continuous	1.67 (1.43, 1.94)	1.63 (1.40, 1.90)	1.66 (1.31, 2.09)
	Quartile 1	1.00 (reference)	1.00 (reference)	1.00 (reference)
	Quartile 2	0.92 (0.55, 1.55)	0.93 (0.56, 1.55)	0.82 (0.38, 1.78)
	Quartile 3	0.97 (0.58, 1.63)	0.96 (0.57, 1.61)	0.85 (0.37, 1.93)
	Quartile 4	2.69 (1.71, 4.22)	2.58 (1.64, 4.06)	2.15 (1.06, 4.33)
	*P* for trend	< 0.001	< 0.001	< 0.001
2&3-PHE	Continuous	1.23 (0.93, 1.64)	1.29 (0.96, 1.73)	1.33 (0.86, 2.01)
	Quartile 1	1.00 (reference)	1.00 (reference)	1.00 (reference)
	Quartile 2	1.08 (0.67, 1.77)	1.09 (0.67, 1.76)	1.04 (0.50, 2.17)
	Quartile 3	1.12 (0.69, 1.81)	1.11 (0.69, 1.81)	1.05 (0.50, 2.21)
	Quartile 4	1.36 (0.85, 2.18)	1.36 (0.85, 2.18)	1.36 (0.66, 2.71)
	*P* for trend	0.149	0.097	0.217

AAC, abdominal aortic calcification; OH-PAHs, mono hydroxylated metabolites of polycyclic aromatic hydrocarbons; 1-NAP, 1-hydroxynaphthalene; 2-NAP, 2-hydroxynaphthalene; 3-FLU, 3-hydroxyfluorene; 2-FLU, 2-hydroxyfluorene; 1-PHE, 1-hydroxyphenanthrene; 1-PYR, 1-hydroxypyrene; 2 & 3-PHE, 2 & 3-hydroxyphenanthrene

Model 1 is unadjusted

Model 2 includes adjustment for age, gender and race

Model 3 includes the variables in model 2 and BMI, education, smoking category, alcohol use, family income, hypertension, diabetes, triglycerides, total cholesterol and cotinine levels

### Subgroup analysis

Subgroup analysis by gender, age, BMI, race, diabetes and hypertension status were performed to further investigate the association between urinary OH-PAHs and severe AAC (Table 3). Subgroup analysis showed that there were no significant interactions with gender, age, BMI, race, diabetes and hypertension status for the association between urinary OH-PAHs and severe AAC (all *P* for interaction > 0.05). The significant associations between urinary OH-PAHs and severe AAC were consistent for subgroups according to gender, age, BMI, race, diabetes and hypertension status.

**Table 3:** ORs and 95% CIs for severe AAC according to quartiles of urinary OH-PAHs, stratified by gender, age, BMI, race, hypertension and diabetes, NHANES 2013–2014^a^

Subgroup	1-NAP	2-NAP	3-FLU	2-FLU	1-PHE	1-PYR	2 & 3-PHE
Gender							
Male	1.06 (1.01, 1.11)	1.39 (1.13, 1.72)	1.08 (0.84, 1.41)	0.97 (0.52, 1.84)	1.06 (0.48, 2.29)	1.53 (1.09, 2.10)	1.19 (0.66, 2.12)
Female	1.07 (1.03, 1.11)	1.37 (1.06, 1.68)	1.19 (0.81, 1.75)	1.32 (0.74, 2.40)	1.13 (0.81, 1.57)	1.90 (1.28, 2.85)	1.45 (0.75, 2.84)
*P* for interaction	0.941	0.945	0.771	0.515	0.860	0.302	0.776
Age (years)							
< 60	1.05 (0.98, 1.14)	1.08 (0.77, 1.50)	0.95 (0.62, 1.31)	0.82 (0.26, 2.65)	1.08 (0.76, 1.55)	1.47 (0.92, 2.30)	1.09 (0.58, 2.05)
≥ 60	1.07 (1.03, 1.11)	1.45 (1.20, 1.79)	1.10 (0.83, 1.45)	2.08 (1.20, 3.62)	1.21 (0.81, 2.62)	1.76 (1.28, 2.41)	1.76 (0.92, 3.37)
*P* for interaction	0.727	0.158	0.216	0.532	0.559	0.371	0.276
BMI (kg/m^2^)							
< 25	1.07 (1.03, 1.14)	1.43 (1.08, 1.89)	1.12 (0.78, 1.58)	1.28 (0.75, 2.24)	1.11 (0.65. 1.88)	1.66 (1.15, 2.37)	1.31 (0.65, 2.71)
25–30	1.05 (0.99, 1.11)	1.25 (0.92, 1.68)	1.01 (0.39, 2.57)	0.92 (0.29, 2.60)	1.00 (0.52, 1.91)	1.54 (0.88, 2.60)	1.42 (0.64, 3.21)
> 30	1.07 (1.01, 1.14)	1.32 (1.04, 1.67)	1.09 (0.82, 1.47)	1.05 (0.37, 2.91)	1.61 (0.73, 3.59)	1.67 (1.06, 2.64)	1.03 (0.38, 2.75)
*P* for interaction	0.862	0.831	0.856	0.722	0.570	0.927	0.889
RACE							
Mexican American	1.05 (0.98, 1.14)	1.55 (0.76, 3.17)	1.03 (0.34, 3.11)	0.72 (0.11, 3.76)	0.91 (0.46, 1.79)	1.40 (0.49, 3.94)	1.88 (0.70, 4.78)
Other Hispanic	1.02 (0.74, 1.42)	0.94 (0.33, 2.66)	1.01 (0.59, 1.75)	0.93 (0.17, 5.19)	1.11 (0.22, 5.85)	1.48 (0.54, 4.03)	1.44 (0.48, 4.30)
Non-Hispanic White	1.08 (1.04, 1.15)	1.40 (1.14, 1.71)	1.14 (0.87, 1.49)	1.59 (0.88, 2.91)	2.05 (0.96, 4.37)	1.91 (1.30, 2.76)	1.16 (0.36, 3.58)
Non-Hispanic Black	1.07 (0.93, 1.23)	1.11 (0.68, 1.80)	0.91 (1.64, 4.92)	0.74 (0.15, 3.60)	1.04 (0.54, 2.02)	1.48 (0.81, 2.72)	0.94 (0.20, 4.46)
Other Race	1.11 (0.97, 1.28)	1.29 (0.80, 2.07)	1.52 (0.48, 4.88)	0.52 (0.06, 5.33)	0.64 (0.12, 3.66)	1.23 (0.44, 3.10)	0.93 (0.26, 3.42)
*P* for interaction	0.830	0.643	0.863	0.738	0.595	0.804	0.852
Hypertension							
No	1.06 (1.02, 1.19)	1.36 (1.08, 1.81)	1.17 (0.92, 1.48)	1.03 (0.56, 1.88)	1.05 (0.74, 1.51)	1.60 (1.19, 2.24)	1.25 (0.85, 2.37)
Yes	1.05 (1.01, 1.16)	1.30 (1.04, 1.69)	0.98 (0.61, 1.57)	1.21 (0.65, 2.24)	1.37 (0.63, 2.85)	1.74 (1.35, 2.24)	1.29 (0.64, 2.62)
*P* for interaction	0.849	0.655	0.528	0.791	0.709	0.896	0.890
Diabetes							
No	1.06 (0.99, 1.13)	1.32 (0.99, 1.76)	0.96 (0.52, 1.79)	1.01 (0.30, 3.51)	1.02 (0.60, 1.82)	1.33 (0.87, 2.01)	1.09 (0.58, 2.06)
Yes	1.07 (1.03, 1.10)	1.35 (1.13, 1.61)	1.14 (0.92, 1.41)	1.12 (0.73, 1.72)	1.15 (0.78, 1.69)	1.89 (1.33, 2.65)	1.82 (0.97, 3.48)
*P* for interaction	0.798	0.609	0.453	0.575	0.578	0.096	0.131

AAC, abdominal aortic calcification; BMI, body mass index; OH-PAHs, mono hydroxylated metabolites of polycyclic aromatic hydrocarbons

Model is adjusted for age, gender and race, BMI, education, smoking category, alcohol use, family income, hypertension, diabetes, cotinine, triglycerides and TC levels

^a^Data are presented as OR (95% CI) unless otherwise indicated

## Discussion

PAHs exposure have been considered as risk factors for cardiovascular diseases (CVD), however, the relationship between PAHs exposure and severe AAC risk is currently lacking. In order to characterize PAHs exposure more comprehensively, we studied several biomarkers of PAHs and found that urinary 1-NAP, 2-NAP and 1-PYR were positively associated with severe AAC prevalence in adults. Therefore, the present study adds an important part to existing literature that higher PAHs exposure are associated with increased risk of severe AAC in adults.

PAHs exposure have been demonstrated to increase the risk of hypertension, peripheral arterial disease (PAD) [29], coronary heart disease (CAD) [30, 31], myocardial infarction (MI) [32] and stroke. A cohort of 12,367 workers from different countries has recorded evidence that the Benzo[a]pyrene (B[a]P) exposure indices were positively related with mortality from ischemic heart disease [18]. Another cohort study also documented that PAHs exposure may increases the risk of mortality from ischemic heart disease [33]. A multi-provincial cohort study revealed that PAHs exposure increase the 10-year risk of atherosclerosis [34]. However, the association between severe AAC and PAHs remains unclear. The present study evaluated this association and found higher PAHs exposure were more likely related to an increased severe AAC prevalence.

However, the mechanisms linking PAHs exposure and AAC remain unclear. Here, we present some potential mechanisms behind the association between PAHs exposure and severe AAC risk. First, inflammation has been well documented as a risk factor for the development of vascular calcification [35, 36]. The association between PAHs and inflammation was supported by population-based studies [37, 38]. In vitro and animal studies have also confirmed that PAHs exposure is positively correlated with systemic inflammation[39]. More importantly, mechanisms that promote the progression of atherosclerosis tend to increase the risk of calcification [40].

Since the composition of PAHs mixtures are not always constant and very complicated in different environments, we studied several biomarkers of PAHs including 1-NAP, 2-NAP, 2-FLU, 3-FLU, 1-PHE, 2&3-PHE, and 1-PYR. In our study, we found that only 1-NAP, 2-NAP and 1-PYR presents positive correlation with the risk of severe AAC prevalence. Studies have shown that 1-PYR is widely used as a biological indicator of PAH exposure [41, 42]. Exposure to 1-PYR increases methylated DNA damage [43]. 1-NAP and 2-NAP are known as metabolites of carbaryl exposure while 1-NAP is also from naphthalene exposure [44]. 1-NAP and 2-NAP exposure increased the prevalence ratio of metabolic syndrome [45, 46]. The present study adds an important part to existing literature that higher PAHs exposure is associated with increased risk of severe AAC in adults. This study provides preliminary evidence of the important relationship between PAHs exposure and severe AAC. More probing research about potential mechanisms is needed in further study.

Our study is based on NHANES database which includes large population of different race, educational backgrounds and epidemiological data, and assess the association between urinary PAHs and severe AAC prevalence. This study still has some limitations. First, causation validation is absent in our cross-sectional study. Second, although we have adjusted some potential confounding factors, the possible impact of unmeasured confounding factors on the relationship between PAHs and severe AAC remains.

## Conclusion

The current study found that urinary 1-NAP, 2-NAP and 1-PYR was positively associated with severe AAC prevalence in adults.

## Data Availability

The datasets presented in this study can be found in NHANES, which is global and public online repositories. The data of this study was available at: https://wwwn.cdc.gov/nchs/nhanes/continuousnhanes/default.aspx?BeginYear=2013.

## Abbreviations

PAHs: Polycyclic aromatic hydrocarbons
AAC: Abdominal aortic calcification
NHANES: National Health and Nutrition Examination Survey
OH-PAHs: Mono hydroxylated metabolites of PAHs
1-NAP: 1-Hydroxynaphthalene
2-NAP: 2-Hydroxynaphthalene
3-FLU: 3-Hydroxyfluorene
2-FLU: 2-Hydroxyfluorene
1-PHE: 1-Hydroxyphenanthrene
1-PYR: 1-Hydroxypyrene
2 & 3-PHE: 2 & 3-Hydroxyphenanthrene
MEC: Mobile examination center
TC: Total cholesterol
B[a]P: Benzo[a]pyrene
